# Comparing the Robustness of ResNet, Swin-Transformer, and MLP-Mixer under Unique Distribution Shifts in Fundus Images

**DOI:** 10.3390/bioengineering10121383

**Published:** 2023-12-01

**Authors:** Kazuaki Ishihara, Koutarou Matsumoto

**Affiliations:** Biostatistics Center, Kurume University, Kurume 830-0011, Japan; a222ms002i@std.kurume-u.ac.jp

**Keywords:** calibration, diabetic retinopathy, distribution shift, fundus image, robustness

## Abstract

Background: Diabetic retinopathy (DR) is the leading cause of visual impairment and blindness. Consequently, numerous deep learning models have been developed for the early detection of DR. Safety-critical applications employed in medical diagnosis must be robust to distribution shifts. Previous studies have focused on model performance under distribution shifts using natural image datasets such as ImageNet, CIFAR-10, and SVHN. However, there is a lack of research specifically investigating the performance using medical image datasets. To address this gap, we investigated trends under distribution shifts using fundus image datasets. Methods: We used the EyePACS dataset for DR diagnosis, introduced noise specific to fundus images, and evaluated the performance of ResNet, Swin-Transformer, and MLP-Mixer models under a distribution shift. The discriminative ability was evaluated using the Area Under the Receiver Operating Characteristic curve (ROC-AUC), while the calibration ability was evaluated using the monotonic sweep calibration error (ECE sweep). Results: Swin-Transformer exhibited a higher ROC-AUC than ResNet under all types of noise and displayed a smaller reduction in the ROC-AUC due to noise. ECE sweep did not show a consistent trend across different model architectures. Conclusions: Swin-Transformer consistently demonstrated superior discrimination compared to ResNet. This trend persisted even under unique distribution shifts in the fundus images.

## 1. Introduction

### 1.1. Background

Diabetes is rapidly increasing worldwide, affecting an estimated 537 million people [1]. Approximately 40–45% of people with diabetes are likely to develop diabetic retinopathy (DR) during their lifetime, a leading cause of visual impairment and blindness [2]. It is important to regularly screen patients with diabetes because early symptoms of DR can be subtle and go unnoticed. Early detection of DR can halt its progression; however, manual diagnosis by ophthalmologists is time-consuming and costly. In addition, there is a shortage of ophthalmologists as the number of diabetes cases increases every year, especially in poor regions such as developing countries. To address these issues, automated screening technologies have received considerable attention, and several deep learning models have been developed to detect DR [2,3,4,5].

The models used in safety-critical applications, such as medical diagnostic devices, must be both discriminative and well calibrated. A model is well calibrated when its output reflects the true correctness likelihood. Recent studies have shown that modern deep learning models are highly discriminative but poorly calibrated [6,7]. Because safety-critical applications make decisions based on the confidence score of the model, overconfidence and underconfidence are significantly detrimental to patients.

In addition, it is critical that the models used in safety-critical applications are robust to distribution shifts where the distributions of the training and test data differ. Distribution shifts can occur naturally in different real-world settings and are influenced by factors such as different hospitals, cameras, or lighting conditions. Previous studies have shown that although deep learning models are highly accurate when the distributions of the training and test data are the same, they can significantly underperform under distribution shifts [8,9]. Therefore, it is extremely important to evaluate models under distribution shifts assumed to occur in real-world settings.

### 1.2. Related Works

#### 1.2.1. Discrimination and Calibration Abilities of Deep Learning Models

There have been many reports on the discrimination and calibration capabilities of deep learning models [7,10,11,12,13,14]. Some studies have suggested that modern high-capacity neural networks, such as ResNet, become overconfident by overfitting to a negative loglikelihood (NLL) [7,15]. In contrast, more modern neural networks with non-convolutional architectures, such as the Vision-Transformer (ViT) and MLP-Mixer, have been reported to possess superior discriminative and calibration abilities [10]. Reportedly, the model size and pre-training scale do not fully explain calibration trends and the model architecture is a critical determinant of calibration [10].

#### 1.2.2. Robustness of Deep Learning Models

In recent years, many studies have investigated the robustness of deep learning models, particularly convolutional neural networks (CNNs) and Transformer-based models. While one study suggested that the robustness of CNNs and ViTs is comparable [16], many studies have reported that ViTs are more robust than CNNs [17,18,19,20]. One reason for the robustness of ViT is that it has a strong shape bias and is similar to the human cognitive system. Therefore, ViT is expected to have better generalizability than CNNs under distributional shifts [19,20,21]. The robustness of MLP-Mixers has been inconclusive, with one study suggesting that MLP-Mixers are as robust as CNNs and another suggesting that MLP-Mixers are superior to CNNs [10,22].

#### 1.2.3. Distribution Shift of Fundus Image

In clinical settings, several factors such as lighting conditions, unexpected eye movements, and ocular lesions including cataracts can affect the quality of fundus images, resulting in uneven illumination, blurring, and low contrast. The degradation of fundus images can affect the diagnosis of DR.

Common image corruptions, including Gaussian noise, snow, frost, brightness, and contrast, are often used to induce distribution shifts in natural image datasets [23]. However, there are concerns regarding the application of these image corruptions to fundus images because of the unique noise that occurs in fundus images.

### 1.3. Objective

Several of the datasets used to investigate model performance under distribution shifts are natural image datasets, such as ImageNet, CIFAR-10, and SVHN, and there is a lack of research investigating model performance under distribution shifts using medical datasets. In this study, we used the retinal fundus image dataset EyePACS [24] to diagnose DR. The purpose of this study was to verify whether the previously reported trends in model performance under distribution shifts remain consistent under unique distribution shifts in fundus images.

## 2. Materials and Methods

### 2.1. Dataset

In this study, we used the open-source DR database EyePACS, which contains 35,126 fundus images of both eyes from different racial backgrounds. We obtained permission from the EyePACS office to access and use the dataset for research purposes (Supplementary Materials).

### 2.2. Outcome

Each image was labeled with DR severity levels based on the International Classification of Diabetic Retinopathy (ICDR) scale. The ICDR scale categorizes DR based on the presence of new blood vessels and distinguishes between non-proliferative diabetic retinopathy (NPDR) and proliferative diabetic retinopathy (PDR). Within NPDR, there are further subcategories: mild, moderate, and severe. Therefore, the ICDR classifies diabetic retinopathy into five levels of severity: no DR, mild NPDR, moderate NPDR, severe NPDR, and PDR. In our study, we adopted a two-class classification task to predict referable DR and defined referable DR as moderate NPDR, severe NPDR, and PDR [25].

### 2.3. Experimental Pipeline

**Figure 1:**
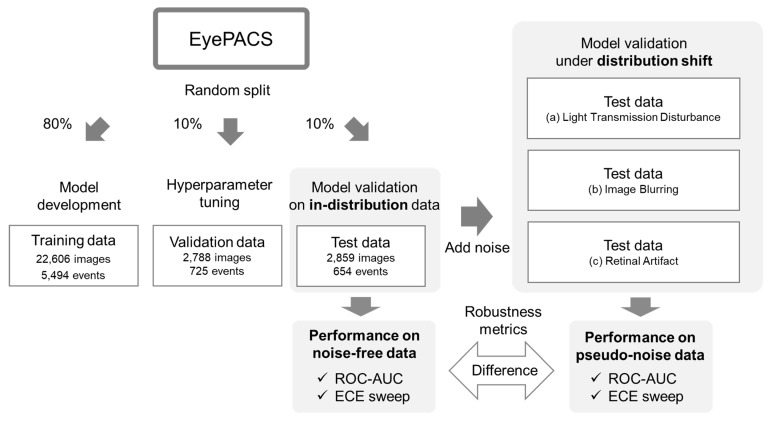
Series of steps from model development to evaluation.

The experimental pipeline is illustrated in Figure 1. The EyePACS database was randomly divided into training (80%), validation (10%), and test (10%) datasets. The training data were used to fine-tune the pre-trained model, the validation data were used to tune hyperparameters such as the number of epochs, and the test data were used to evaluate model performance on in-distribution data and under distribution shifts. In-distribution refers to scenarios in which the fundus image remains unaltered, whereas a distribution shift refers to scenarios in which noise is introduced into the image. Following previous studies, we induced a distribution shift by introducing three types of noise that can occur in real-world settings during fundus imaging examinations [26]. In previous research, the difference in evaluation metrics before and after the addition of noise has been used as a metric of model robustness [22,23]. Therefore, we adopted the same definition in our study. 

### 2.4. Preprocessing

The Benjamin Graham method was used to improve the lighting conditions of the fundus image [27,28]. Subsequently, the images were normalized and resized to 224 × 224 pixels. In addition, random horizontal and rotational magnifications were applied.

### 2.5. Models

To evaluate model robustness, we adopted three model architectures: ResNet, Swin-Transformer, and MLP-Mixer and used pre-trained models (Table 1). The pre-trained models were tuned across all layers. The models were trained for 100 epochs with a batch size of 128. We used 10^−4^ as the base learning rate for the Adam optimizer, along with a default of 20 warm-up iterations and 10^−5^ as the weight decay. During training, the learning rate was reduced by a factor of 10 after 30, 60, and 90 epochs.

1. ResNet [29] is a widely used model with a convolutional structure that incorporates residual connections. We used three ResNets with different model sizes: ResNet-50, ResNet-101, and ResNet-152.
2. Swin-Transformer [30] is a model with a non-convolutional structure that implements a hierarchical structure using shifted windows in the Vision-Transformer [31]. We used four Swin-Transformers with different model sizes: Tiny, Small, Base, and Large.
3. MLP-Mixer [32] is a model implemented using only a multilayer perceptron without a convolutional structure or attention mechanism. We used two MLP-Mixers with different model sizes: Base and Large.

### 2.6. Evaluation

The Area Under the Receiver Operating Characteristic- Curve (ROC-AUC) was used to evaluate the discriminative ability of the models. While the Expected Calibration Error (ECE) is commonly used to evaluate the calibration ability of models [7,33], it has been reported to be an inadequate estimator of calibration error due to its systematic non-negligible bias [34]. Therefore, in this study, we used the monotonic sweep calibration error (ECE sweep) [34], which has been suggested as an estimator with a lower bias than the ECE. The calibration metrics are described in detail below. The robustness of a model was evaluated based on the difference between its performance on noise-free data and that on data with pseudo-noise.

#### Calibration Metrics

We consider a binary classification with input X∈χ, output $Y=\left\{0,1\right\}$, and model $f:X\to \left[0,1\right]$ that predicts the confidence score of the true label Y to be 1. Model f is well calibrated if its output correctly reflects the true correctness likelihood. Formally, a perfectly calibrated model satisfies:

(1) $$\begin{array}{c} P\left(Y=1|f\left(X\right)=p\right)=p,\forall p\;. \end{array}$$

True Calibration Error (TCE) is widely used to measure the calibration error by calculating the expected deviation between both sides of Equation (1).

(2) $$\begin{array}{c} \mathrm{TCE}\left(f\right)=E_{X}\left[\left|f\left(X\right)-E_{Y}\left[Y|f\left(X\right)\right]\right|\right]. \end{array}$$

$f\left(X\right)$ represents the distribution of confidence scores, whereas $E_{Y}\left[Y|f\left(X\right)\right]$ denotes the true calibration curve and illustrates the relationship between the empirical accuracy and confidence scores.

To estimate the TCE of model f, if we are given a finite sample ${\left\{x_{i},y_{i}\right\}}_{i=1}^{n}$, we typically group the sample into equally spaced bins ${\left\{B_{m}\right\}}_{m=1}^{M}$ based on confidence scores and then calculate the expected difference between the average confidence score $\bar{f_{k}}$ and the proportion $\bar{y_{k}}$ where the true label Y is 1.

(3) $$\begin{array}{c} \mathrm{ECE}=\overset{b}{\underset{k=1}{\sum }}\frac{\left|B_{k}\right|}{n}\left|\bar{f_{k}}-\bar{y_{k}}\right|\;. \end{array}$$

The calculation of ECE is known to be sensitive to hyperparameters, such as the chosen binning method and the number of bins [35]. In addition, ECE is an inherently biased estimator, and it has been empirically observed that there exists an optimal number of bins that minimizes estimation bias, which tends to increase with the sample size [34]. To address this and determine the optimal number of bins, an ECE sweep is proposed, assuming a monotonically increasing behavior in the true calibration curve and providing a less-biased estimator [34]. The ECE sweep chooses the largest number of bins that preserve monotonicity in the proportion $\bar{y_{k}}$.

(4) $$\begin{array}{c} \begin{array}{c} {\mathrm{ECE}}_{\mathrm{SWEEP}}=\overset{b^{*}}{\underset{k=1}{\sum }}\frac{\left|B_{k}\right|}{n}\left|\bar{f_{k}}-\bar{y_{k}}\right|\;\mathrm{where}\\ b^{*}=\max\left\{b|1\leq b\leq n;\forall b^{\prime }\leq b,\bar{y_{1}}\leq \cdots \leq \bar{y_{b^{\prime }}}\right\}.\; \end{array} \end{array}$$

### 2.7. Distribution Shift of Fundus Image

**Figure 2:**
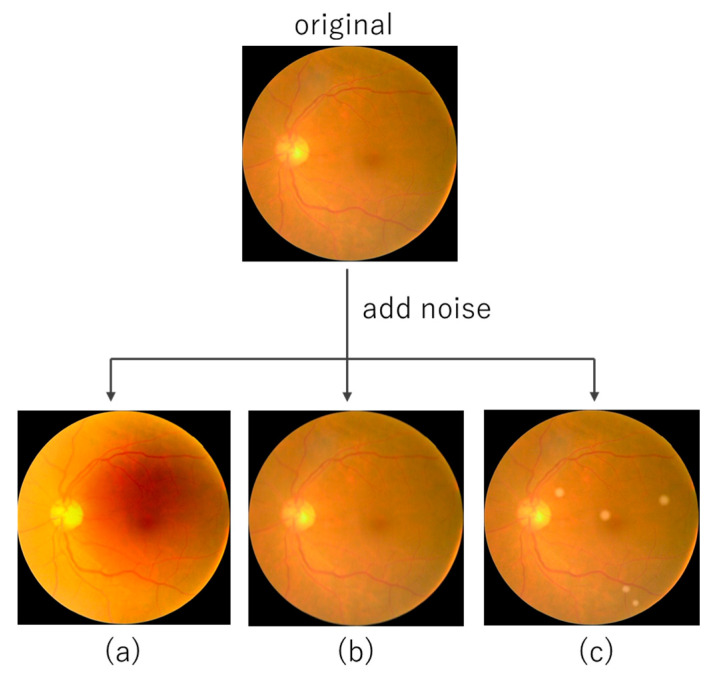
Examples of fundus images without noise and with three types of added noise. (**a**) Light Transmission Disturbance, (**b**) Image Blurring, (**c**) Retinal Artifact.

Various factors, such as lighting conditions, unexpected eye movements, and ocular pathologies, such as cataracts, can cause uneven illumination, blurring, and low contrast. These elements can significantly degrade the quality of the fundus images. Based on the three realistically occurring factors defined by Shen et al. [26]: (a) Light Transmission Disturbance, (b) Image Blurring, and (c) Retinal Artifact, noise was added to the test data to evaluate the robustness of the model under distribution shifts (Figure 2). To facilitate the interpretation of the effect of noise introduced into retinal images on prediction accuracy, three different noise sources were evaluated one at a time.

#### 2.7.1. Light Transmission Disturbance

The fundus camera was programmed for automatic exposure; however, unstable stray light can cause under/over exposure. Differences in the distance between the fundus and ophthalmoscope can cause uneven illumination due to differences in the sensitivity of certain regions of the image plane. To model these factors, the light transmission disturbance is defined for a clean image x and its degraded image $x^{\prime }$ as

$$x^{\prime }=\mathrm{clip}\left(\alpha \left(J\cdot G_{L}\left(r_{L},\sigma_{L}\right)+x\right)+\beta ;s\right),\;$$

where *α*, *β*, and *s* refer to the factors for contrast, brightness, and saturation, respectively. $\mathrm{Clip}\;\left(\beta ;\;s\right)$ represents a clipping function. $G_{L}$ represents a Gaussian kernel. J represents the illumination bias to be over- or under-illuminated in a panel centered at (*a*, *b*) with a radius of $r_{L}$.

#### 2.7.2. Image Blurring

Blurring can be caused by several factors, such as program settings during the fundus imaging procedure, human error, or the presence of cataracts. To model these factors, Image Blurring is defined for a clean image x and its degraded image $x^{\prime }$ as

$$x^{\prime }=x\cdot G_{B}\left(r_{B},\sigma_{B}\right)+n,$$

where $G_{B}$ is a Gaussian filter with a radius $r_{B}$ and spatial constant $\sigma_{B}$, and *n* denotes the additive random Gaussian noise.

#### 2.7.3. Retinal Artifact

Imaging in poor conditions can degrade the quality of the fundus image due to dust and grains attached to the lens of the imaging plane. To model these factors, Retinal Artifact is defined for a clean image x and its degraded image $x^{\prime }$ as

$$x^{\prime }=x+\overset{K}{\underset{k}{\sum }}G_{R}\left(r_{k}/4,\sigma_{k}\right)\cdot o_{k},$$

where the Gaussian filter used is $G_{R}$, with a specified radius of $r_{k}$ for object *k* deemed undesirable and its corresponding variance $\sigma_{k}$. The luminance bias is also represented by $o_{k}$.

## 3. Results

### 3.1. Model Performance on In-Distribution Data

**Figure 3:**
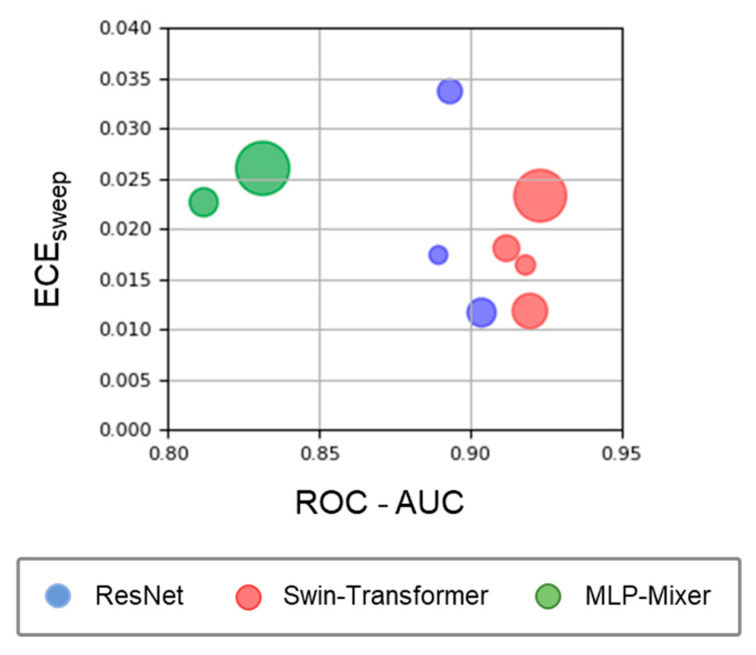
Model performance on in-distribution data. Circle size represents the model size, with the ROC-AUC on the *x*-axis and the ECE sweep on the *y*-axis. The blue, red, and green plots represent the ResNet, Swin-Transformer, and MLP-Mixer architectures, respectively.

First, we assessed the discrimination and calibration ability of the models on in-distribution data (Figure 3). The ROC-AUC was the highest for Swin-Transformer (the lowest and highest values for different model sizes: 0.912–0.923), followed by ResNet (0.889–0.904) and MLP-Mixer (0.812–0.831). No significant differences were found between the model architectures in the ECE sweep (Swin-Transformer: 0.012–0.023, ResNet: 0.012–0.034, MLP-Mixer: 0.023–0.026). For all three model architectures, the model size tended to increase with the ROC-AUC value, but this trend was not found in the ECE sweep.

### 3.2. Model Performance under Distribution Shift

**Figure 4:**
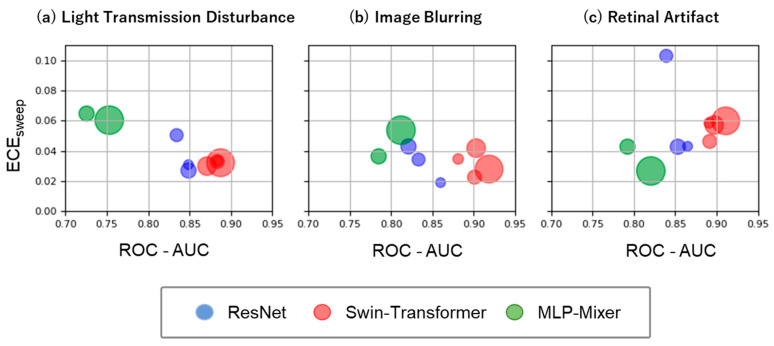
Model performance under three unique distribution shifts in the fundus images: Light Transmission Disturbance (**a**), Image Blurring (**b**), and Retinal Artifact (**c**). Plot details are the same as for Figure 3.

We assessed the discrimination and calibration abilities of the models under three unique distribution shifts in the fundus images (Figure 4). Similar to in-distribution, the ROC-AUC is highest for Swin-Transformer ((a) 0.871–0.887, (b) 0.881–0.918, (c) 0.891–0.911), followed by ResNet ((a) 0.834–0.849, (b) 0.821–0.860, (c) 0.839–0.865) and MLP-Mixer ((a) 0.725–0.753, (b) 0.785–0.812, (c) 0.792–0.820). No consistent trend was found for the ECE sweep as its value for each model differed depending on the distribution shift type and model size (Swin-Transformer: (a) 0.030–0.033, (b) 0.023–0.042, and (c) 0.046–0.060; ResNet: (a) 0.027–0.050, (b) 0.019–0.0430, and (c) 0.043–0.103; MLP-Mixer: (a) 0.060–0.065, (b) 0.036–0.054, and (c) 0.027–0.043).

### 3.3. Difference in Model Performance between Noise-Free and Pseudo-Noise Data

**Figure 5:**
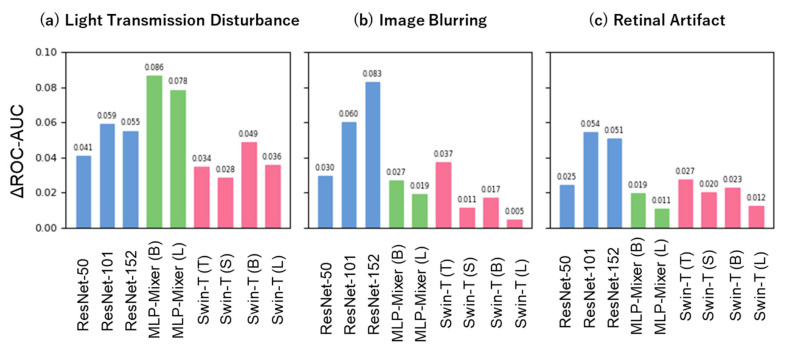
Robustness for discriminative ability under three unique distribution shifts. The *x*-axis showcases each model architecture with different model sizes. The blue, green, and red bars represent the ResNet, MLP-Mixer, and Swin-Transformer architectures, respectively. The *y*-axis highlights the difference in ROC-AUC between distribution shift and in-distribution.

We first assessed the robustness of the models in terms of discriminability (Figure 5). The ROC-AUC difference for the distribution shift caused by Light Transmission Disturbance (Figure 5a), compared to in-distribution, was the smallest for Swin-Transformer (0.028–0.049), followed by ResNet (0.041–0.059) and MLP-Mixer (0.078–0.086). The ROC-AUC difference for the distribution shift caused by Image Blurring (Figure 5b) was comparatively small for both the Swin-Transformer (0.005–0.037) and MLP-Mixer (0.019–0.027), followed by ResNet (0.030–0.083). The ROC-AUC difference for the distribution shift caused by Retinal Artifact (Figure 5c) was the lowest for MLP-Mixer (0.011–0.019), followed by Swin-Transformer (0.012–0.027) and ResNet (0.025–0.054). Compared to ResNet, Swin-Transformer showed a smaller reduction in the ROC-AUC across all noise types. We also compared the ROC-AUC reductions across the three distribution shifts within each model; both the Swin-Transformer and MLP-Mixer tended to deteriorate mainly under the distribution shift caused by Light Transmission Disturbance. From the perspective of model size, ResNet tended to increase the reduction in the ROC-AUC with increasing model size. In contrast, Swin-Transformer and MLP-Mixer tended to decrease the reduction in the ROC-AUC with increasing model size.

**Figure 6:**
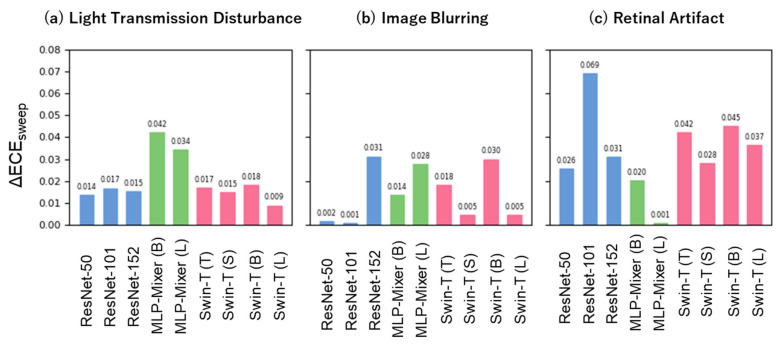
Robustness for calibration ability under three unique distribution shifts. The *x*-axis depicts each model architecture with different model size. The blue, green, and red bars represent the ResNet, MLP-Mixer, and Swin-Transformer architectures, respectively. The *y*-axis represents the difference in ECE sweep between distribution shift and in-distribution.

Next, we assessed the robustness of the models in terms of their calibration ability (Figure 6). The difference in the ECE sweep values between distribution shift and in-distribution did not show a consistent trend as the ECE sweep value for each model varied depending on the type of distribution shift and model size (Swin-Transformer: (a) 0.009–0.018, (b) 0.005–0.030, and (c) 0.028–0.045; ResNet: (a) 0.014–0.017, (b) 0.001–0.031, and (c) 0.026–0.069; MLP-Mixer: (a) 0.034–0.042, (b) 0.014–0.028, and (c) 0.001–0.020). In contrast, when comparing the reduction in the ECE sweep across the three distribution shifts within each model, the MLP-Mixer tended to degrade under the distribution shift caused by Light Transmission Disturbance, whereas both ResNet and Swin-Transformer tended to degrade under the distribution shift caused by Retinal Artifact.

## 4. Discussion

The main findings of this study are as follows: Swin-Transformer displayed a consistently higher discriminative ability than ResNet. This trend persisted even under unique distribution shifts in the fundus images. No significant differences were found in the calibration ability between the model architectures and model sizes.

### 4.1. Model Discrimination and Calibration Ability

Swin-Transformer demonstrated superior discriminative ability under both in-distribution and distribution shifts, followed by ResNet and MLP-Mixer. These results are consistent with those of a previous study using a natural image dataset [22]. Significant findings in retinal images of diabetic retinopathy include capillary aneurysms, beaded expansion, intraretinal microvascular abnormalities, hard exudates, soft exudates, new vessels, and vitreous hemorrhage. These findings are primarily localized and appear at different scales within retinal images. A Swin-Transformer model builds hierarchical feature maps by gradually merging features from adjacent small patches to create representations for larger patches. This hierarchical structure can capture features at different scales ranging from global image features to finer details. This approach may effectively capture the localized and different-scale features of diabetic retinopathy present in retinal images, potentially leading to its high discriminative performance.

Previous studies suggest that non-convolutional models, such as ViT and MLP-Mixer, have a better calibration ability than CNNs in both in-distribution and distribution shifts [10]. In addition, it has been reported that large deep learning models trained with a large number of parameters using negative log-likelihood exhibit overconfidence [7,15]. However, in this study, no significant differences were observed in calibration performance based on model architecture or size. Several previous studies identified factors that affect calibration, including regularization, model size, insufficient data, and imbalanced data [7,12]. As suggested by previous studies, various factors could complexly influence calibration performance, making it challenging to discern differences due to the architecture or model size; therefore, further research is needed.

### 4.2. Model Robustness

Previous studies have suggested that Transformer-based models are more robust than CNNs in their discriminative and calibration abilities [10,17,18,19,20]. Similarly, our study indicates that Swin-Transformer is more robust in its discriminative ability than ResNet as it consistently achieves a smaller reduction in the ROC-AUC across all distribution shifts considered in this study. Previous studies on the robustness of MLP-Mixer compared to CNNs have provided contradicting results [10,22]. Our study could not demonstrate the robustness of MLP-Mixture.

Herein, we considered three types of noise that can occur in fundus images. We hypothesized that Light Transmission Disturbance would primarily affect the texture of images, Image Blurring would affect their shape, and Retinal Artifact would potentially affect both texture and shape. Previous studies have suggested a strong texture bias in CNNs, whereas Transformer-based models, including ViT, indicate a stronger shape bias [19,20,21]. Therefore, we postulated that ResNet might be particularly susceptible to distribution shifts induced by Light Transmission Disturbance and Swin-Transformer to those induced by Image Blurring. However, our findings did not corroborate these anticipated tendencies (Figure 5 and Figure 6). The discrepancy between our assumptions and results could be due to the fact that Light Transmission Disturbances strongly affect not only the texture but also the shape. Alternatively, the low intensity of the image blurring noise could have resulted in a minimal effect on the shape. Further research is needed to draw definitive conclusions.

The calibration ability of ResNet and Swin-Transformer significantly worsened under the distribution shifts caused by Retinal Artifact (Figure 6c). Previous research has suggested that adversarial attacks on medical images are easier to conduct than on natural images, indicating a vulnerability in deep neural network models developed for medical images [36]. This is because medical images have complex biological textures, resulting in regions of high gradients that are sensitive to small adversarial perturbations. Therefore, in our study retinal artifacts may have behaved similarly to adversarial perturbations, potentially influencing the calibration performance.

In addition, MLP-Mixer was particularly susceptible to distribution shifts caused by Light Transmission Disturbance (Figure 5a and Figure 6a), suggesting that it may be affected by distribution shifts based on principles different from those of ResNet and Swin-Transformer, which requires further investigation.

### 4.3. Limitations

This study had several limitations. First, because a single dataset was used, additional verification using different fundus image datasets is required to validate the results of this study across all fundus images. Second, models with lower inductive bias, such as the Swin-Transformer and MLP-Mixer, require large amounts of data to improve accuracy; the dataset used in this study may not be large enough for these models to demonstrate their intrinsic capabilities. To mitigate this problem, we fine-tuned the models that were pre-trained on ImageNet. Finally, the recently developed ConvNeXt, a CNN architecture, was reported to exhibit robustness comparable to that of Transformer-based models [16]. Further research is needed to compare the robustness of convolutional and Transformer-based models.

## 5. Conclusions

In this study, we assessed the performances of the ResNet, Swin-Transformer, and MLP-Mixer models under unique distribution shifts in fundus images. Swin-Transformer demonstrated superior and robust discriminative ability than ResNet under both in-distribution and distribution shifts. In contrast to the previously reported trends in model calibration, this study did not observe significant differences in calibration ability based on model architecture and model size. This discrepancy can be attributed to the unique characteristics of the medical dataset. These findings highlight the need for additional multifaceted validation processes focused on retinal images and additional verification using different fundus image datasets.

## Figures and Tables

**Table 1:** Pre-trained models used in this study.

Model Name	Model Size (Parameters)
ResNet-50	23.5 M
ResNet-101	42.5 M
ResNet-152	58.1 M
Swin-Transformer (Tiny)	27.5 M
Swin-Transformer (Small)	48.8 M
Swin-Transformer (Base)	86.7 M
Swin-Transformer (Large)	195 M
MLP-Mixer (Base)	59.1 M
MLP-Mixer (Large)	207 M

## Data Availability

Restrictions apply to the availability of these data. Data were obtained from Kaggle and are available [https://www.kaggle.com/c/diabetic-retinopathy-detection] (accessed on 22 October 2023) with permission from the EyePACS office.

## Supplementary Materials

Analysis was conducted using Python (version 3.10.11) and PyTorch (version 1.11.0). Further details regarding these programs are available on GitHub (https://github.com/kazuakiishihara/), accessed on 22 October 2023.

## References

[1] International Diabetes Federation (2021). IDF Diabetes Atlas.

[2] Gargeya R., Leng T. (2017). Automated Identification of Diabetic Retinopathy Using Deep Learning. Ophthalmology.

[3] Gulshan V., Peng L., Coram M., Stumpe M.C., Wu D., Narayanaswamy A., Venugopalan S., Widner K., Madams T., Cuadros J. (2016). Development and validation of a deep learning algorithm for detection of diabetic retinopathy in retinal fundus photographs. JAMA.

[4] Alyoubi W.L., Abulkhair M.F., Shalash W.M. (2021). Diabetic retinopathy fundus image classification and lesions localization system using deep learning. Sensors.

[5] Pires R., Avila S., Wainer J., Valle E., Abramoff M.D., Rocha A. (2019). A data-driven approach to referable diabetic retinopathy detection. Artif. Intell. Med..

[6] Hein M., Andriushchenko M., Bitterwolf J. Why ReLU networks yield high-confidence predictions far away from the training data and how to mitigate the problem. Proceedings of the Computer Vision and Pattern Recognition.

[7] Guo C., Pleiss G., Sun Y., Weinberger Q.K. On calibration of modern neural networks. Proceedings of the International Conference on Machine Learning.

[8] Taori R., Dave A., Shankar V., Carlini N., Recht B., Schmidt L. Measuring robustness to natural distribution shifts in image classification. Proceedings of the Neural Information Processing Systems.

[9] Recht B., Roelofs R., Schmidt L., Shankar V., ImageNet D. Classifiers generalize to ImageNet?. Proceedings of the International Conference on Machine Learning.

[10] Minderer M., Djolonga J., Romijnders R., Hubis F., Zhai X., Houlsby N., Tran D., Lucic M. Revisiting the calibration of modern neural networks. Proceedings of the Neural Information Processing Systems.

[11] Ovadia Y., Fertig E., Ren J., Nado Z., Sculley D., Nowozin S., Dillon J., Lakshminarayanan B., Snoek J. Can you trust your Model’s uncertainty? Evaluating predictive uncertainty under dataset shift. Proceedings of the Neural Information Processing Systems.

[12] Wang D., Feng L., Zhang M.L. Rethinking calibration of deep neural networks: Do not be afraid of overconfidence. Proceedings of the Neural Information Processing Systems.

[13] Krishnan R., Tickoo O. Improving model calibration with accuracy versus uncertainty optimization. Proceedings of the Neural Information Processing Systems.

[14] Karandikar A., Cain N., Tran D., Lakshminarayanan B., Shlens J., Mozer M.C., Roelofs B. Soft calibration objectives for neural networks. Proceedings of the Neural Information Processing Systems.

[15] Mukhoti J., Kulharia V., Sanyal A., Golodetz S., Torr P., Dokania P. Calibrating deep neural networks using focal loss. Proceedings of the Neural Information Processing Systems.

[16] Pinto F., Torr P., Dokania P. An impartial take to the CNN vs transformer robustness contest. Proceedings of the European Conference on Computer Vision.

[17] Paul S., Chen P. Vision transformers are robust learners. Proceedings of the AAAI Conference on Artificial Intelligence.

[18] Bhojanapalli S., Chakrabarti A., Glasner D., Li D., Unterthiner T., Veit A. Understanding robustness of transformers for image classification. Proceedings of the International Conference on Computer Vision.

[19] Zhang C., Zhang M., Zhang S., Jin D., Zhou Q., Cai Z., Zhao H., Liu X., Liu Z. Delving deep into the generalization of vision transformers under distribution shifts. Proceedings of the Computer Vision and Pattern Recognition.

[20] Naseer M., Ranasinghe K., Khan S., Hayat M., Khan F., Yang M. Intriguing properties of vision transformers. Proceedings of the Neural Information Processing Systems.

[21] Geirhos R., Rubisch P., Michaelis C., Bethge M., Wichmann F., Brendel W. ImageNet-trained CNNs are biased towards texture; increasing shape bias improves accuracy and robustness. Proceedings of the International Conference on Learning Representations.

[22] Morrison K., Gilby B., Lipchak C., Mattioli A., Kovashka A. Exploring corruption robustness: Inductive biases in vision transformers and MLP-mixers. Proceedings of the International Conference on Machine Learning.

[23] Hendrycks D., Dietterich T. Benchmarking neural network robustness to common corruptions and perturbations. Proceedings of the International Conference on Learning Representations.

[24] Cuadros J., Bresnick G. (2009). EyePACS: An adaptable telemedicine system for diabetic retinopathy screening. J. Diabetes Sci. Technol..

[25] Ghanchi F., Bailey C., Chakravarthy U., Cohen S., Dodson P., Gibson J., Menon G., Muqit M., Pilling R., Olson J. (2012). Diabetic Retinopathy Guidelines. https://www.rcophth.ac.uk/wp-content/uploads/2021/08/2012-SCI-267-Diabetic-Retinopathy-Guidelines-December-2012.pdf.

[26] Shen Z., Fu H., Shen J., Shao L. (2021). Modeling and enhancing low-quality retinal fundus images. IEEE Trans. Med. Imaging.

[27] Ratthachat C. (2019). APTOS: Eye Preprocessing in Diabetic Retinopathy (Kaggke Report). https://www.kaggle.com/code/ratthachat/aptos-eye-preprocessing-in-diabetic-retinopathy/comments.

[28] Graham B. (2015). Kaggle Diabetic Retinopathy Detection Competition Report. https://www.kaggle.com/c/diabetic-retinopathy-detection.

[29] He K., Zhang X., Ren S., Sun J. Deep residual learning for image recognition. Proceedings of the Computer Vision and Pattern Recognition.

[30] Liu Z., Lin Y., Cao Y., Hu H., Wei Y., Zhang Z., Lin S., Guo B. Swin transformer: Hierarchical vision transformer using shifted windows. Proceedings of the International Conference on Computer Vision.

[31] Dosovitskiy A., Beyer L., Kolesnikov A., Weissenborn D., Zhai X., Unterthiner T., Dehghani M., Minderer M., Heigold G., Gelly S. An image is worth 16x16 words: Transformers for image recognition at scale. Proceedings of the International Conference on Learning Representations.

[32] Tolstikhin I.O., Houlsby N., Kolesnikov A., Beyer L., Zhai X., Unterthiner T., Yung J., Steiner A., Keysers D., Uszkoreit J. MLP-Mixer: An all-MLP architecture for vision. Proceedings of the Neural Information Processing Systems.

[33] Naeini M.P., Cooper G., Hauskrecht M. Obtaining Well Calibrated Probabilities Using Bayesian Binning. Proceedings of the Twenty-Ninth AAAI Conference on Artificial Intelligence.

[34] Roelofs R., Cain N., Shlens J., Mozer M.C. Mitigating bias in calibration error estimation. Proceedings of the International Conference on Artificial Intelligence and Statistics.

[35] Nixon J., Dusenberry M.W., Zhang L., Jerfel G., Tran D. Measuring calibration in deep learning. Proceedings of the Computer Vision and Pattern Recognition.

[36] Ma X., Niu Y., Gu L., Wang Y., Zhao Y., Bailey J., Lu F. (2021). Understanding adversarial attacks on deep learning based medical image analysis systems. Pattern Recognit..

